# Supplementary material for: Structure of the mature Rous sarcoma virus lattice reveals a role for IP6 in the formation of the capsid hexamer
Source: Nat Commun. 2021 May 28;12:3226. doi: 10.1038/s41467-021-23506-0 (PMC8163826; doi:10.1038/s41467-021-23506-0)
Supplement: Supplementary file 1 — Supplementary Information [file 41467_2021_23506_MOESM1_ESM.pdf]

Supplementary Information

Structure of the mature Rous sarcoma virus lattice reveals a role for IP6 in the formation of the capsid hexamer

Martin Obr[1], Clifton L. Ricana[2], Nadia Nikulin[3], Jon-Philip R. Feathers[3], Marco Klanschnig[1], Andreas Thader[1], Marc C. Johnson[2], Volker M. Vogt[3], Florian K.M. Schur[1#], Robert A. Dick[3#]

1. Institute of Science and Technology Austria, Klosterneuburg, Austria
2. Department of Molecular Microbiology and Immunology, University of Missouri, Columbia, MO
3. Department of Molecular Biology and Genetics, Cornell University, Ithaca, NY

[#] Correspondence to: florian.schur@ist.ac.at (FKMS), rad82@cornell.edu (RAD)

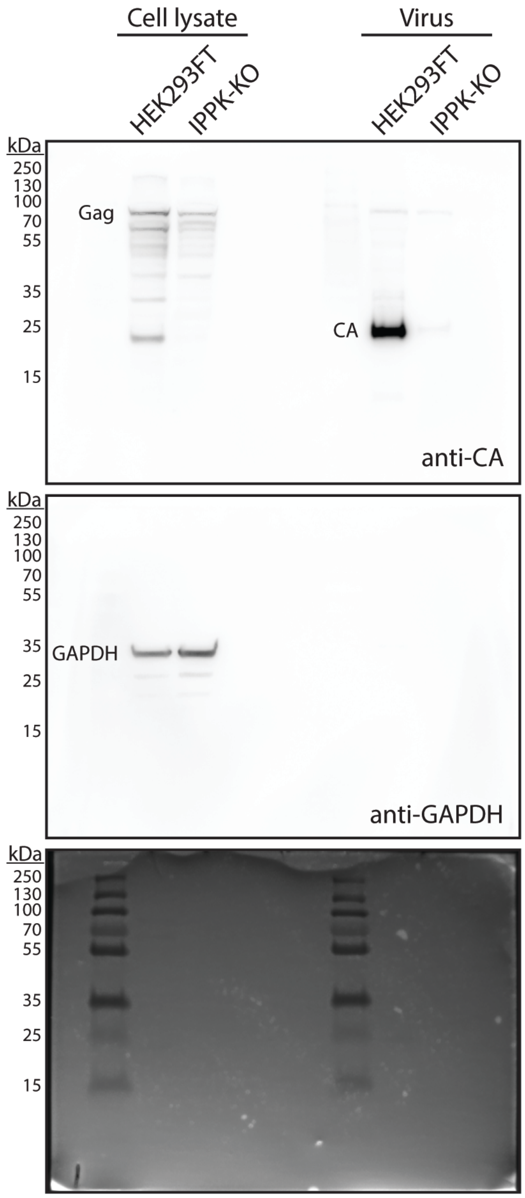

Supplementary Figure 1. Representative western blots. Uncropped western blots corresponding to the cropped blots in Fig. 1e. (Top) Gag expression and release of virus from WT HEK293FT and IPPK-KO cells, detected by rabbit anti-CA antibody. Bands that were quantified are full-length Gag and CA, as labeled. (Middle) Same membrane as in top, stripped, and re-blotted with mouse anti-GAPDH. (Bottom) Same membrane as in top and middle, showing molecular weight ladder.

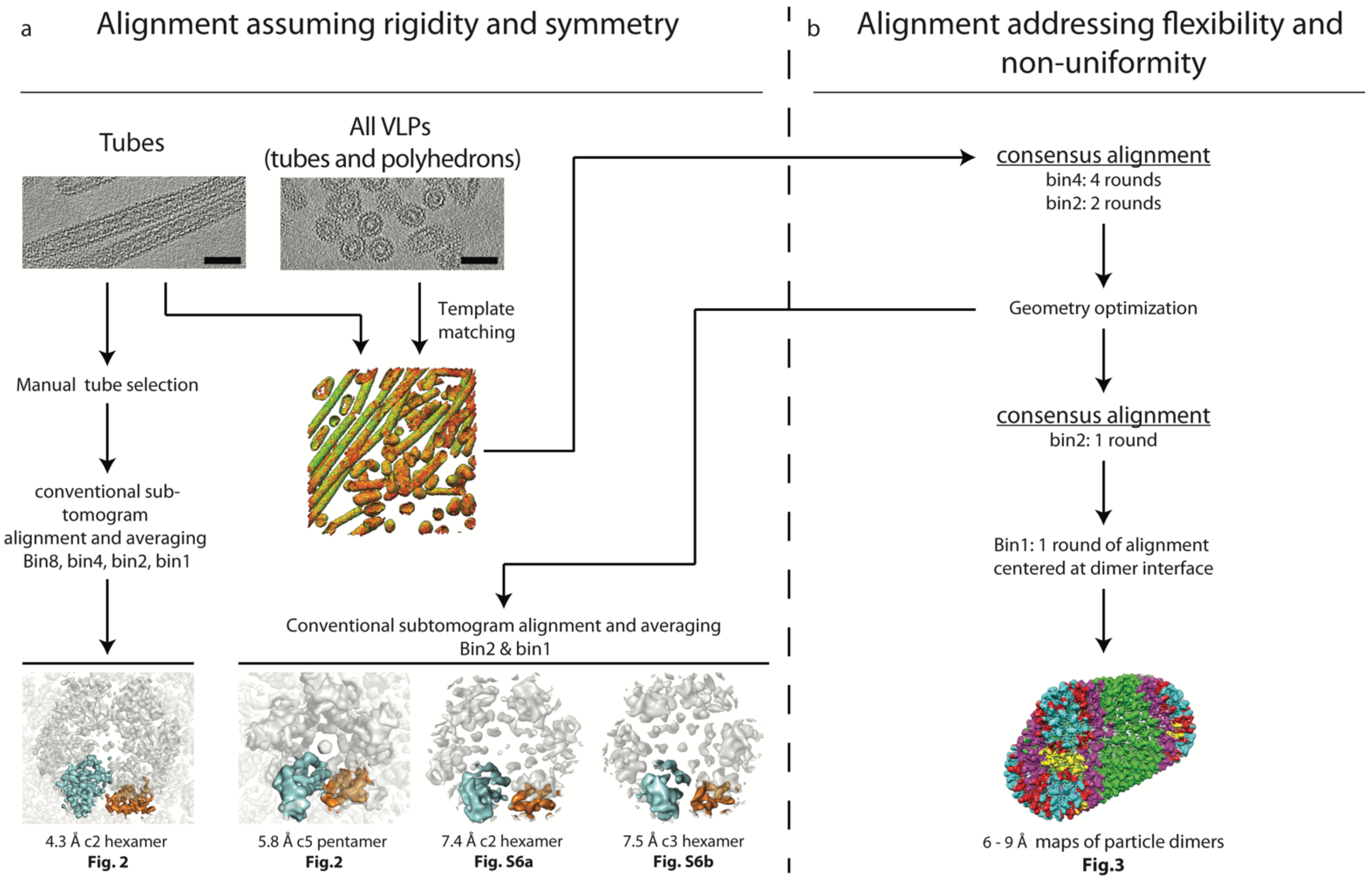

Supplementary Figure 2: Subtomogram averaging workflows. Basic schematic summary of the subtomogram averaging workflows employed in this study. **a)** On the left side the standard subtomogram alignment assuming rigidity and symmetry is shown. Scale bars in the slices from cryo-electron tomograms are 50nm. The slices are representative of the 49 tomogram dataset. **b)** The parts of the workflow using the novel consensus alignment routines are shown on the right side. Connections between the two approaches, underlining their modularity, are annotated by arrows. Details on the novel alignment and classification strategy are given in Methods and Supplementary Fig. 5.

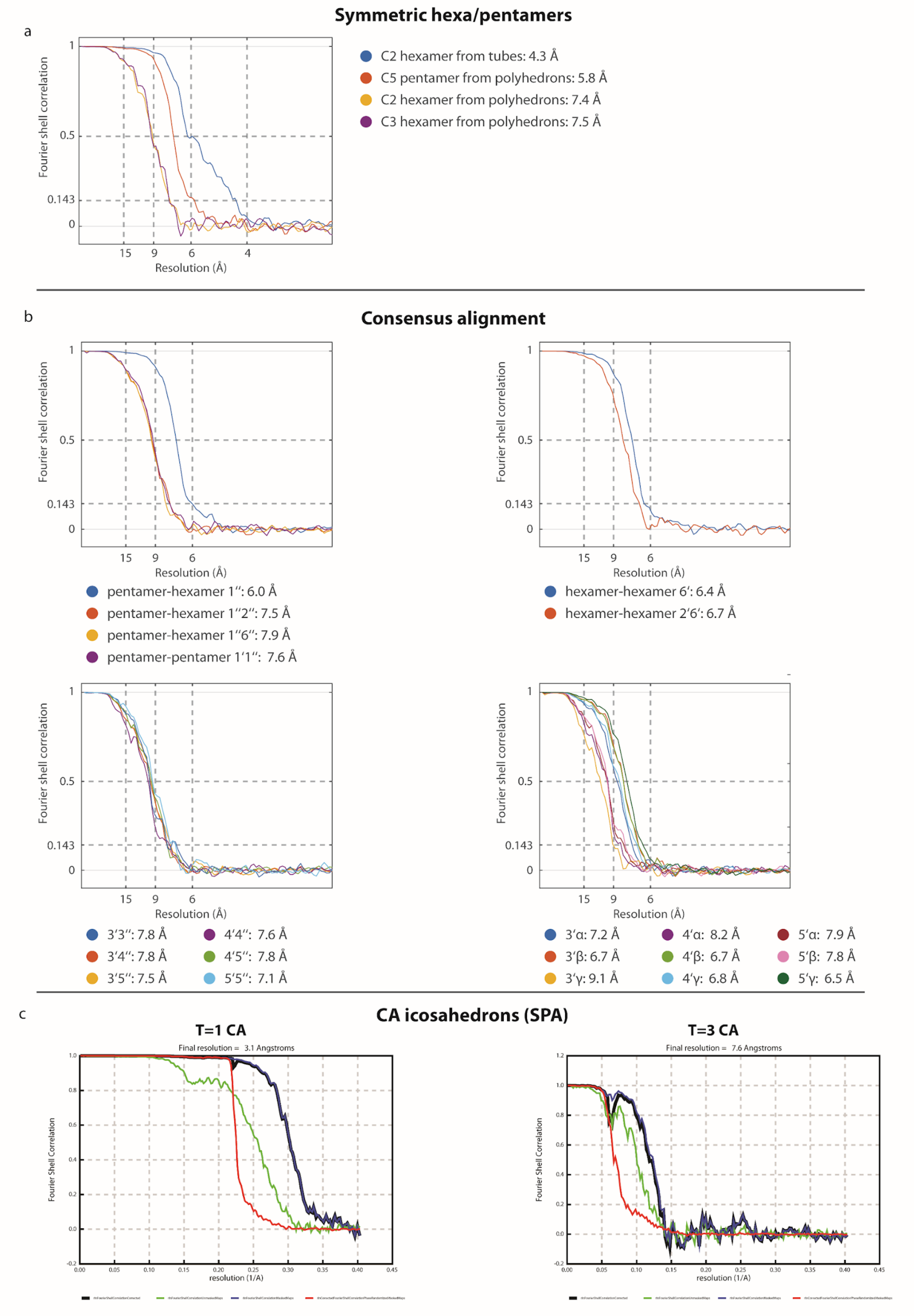
a
Symmetric hexa/pentamers
Fourier shell correlation
1
0.5
0.143
0
15
9
6
4
Resolution (Å)
C2 hexamer from tubes: 4.3 Å
C5 pentamer from polyhedrons: 5.8 Å
C2 hexamer from polyhedrons: 7.4 Å
C3 hexamer from polyhedrons: 7.5 Å
b
Consensus alignment
Fourier shell correlation
Resolution (Å)
pentamer-hexamer 1'': 6.0 Å
pentamer-hexamer 1''2'': 7.5 Å
pentamer-hexamer 1''6'': 7.9 Å
pentamer-pentamer 1'1'': 7.6 Å
hexamer-hexamer 6': 6.4 Å
hexamer-hexamer 2'6': 6.7 Å
3'3'': 7.8 Å
3'4'': 7.8 Å
3'5'': 7.5 Å
4'4'': 7.6 Å
4'5'': 7.8 Å
5'5'': 7.1 Å
3'α: 7.2 Å
3'β: 6.7 Å
3'γ: 9.1 Å
4'α: 8.2 Å
4'β: 6.7 Å
4'γ: 6.8 Å
5'α: 7.9 Å
5'β: 7.8 Å
5'γ: 6.5 Å
c
CA icosahedrons (SPA)
T=1 CA
Final resolution = 3.1 Angstroms
Fourier Shell Correlation
resolution (1/A)
T=3 CA
Final resolution = 7.6 Angstroms
Fourier Shell Correlation
resolution (1/A)

Supplementary Figure 3: Resolution estimation via Fourier Shell correlation. a) FSC curves for pentamers and hexamers solved by subtomogram averaging, while utilizing the corresponding symmetries (C2, C3, C5). **b)** FSC curves for different classes from groups I-IV, which were analyzed using consensus alignment. **c)** FSC curves for RSV CA icosahedrons analyzed by single-particle analysis. The number of subvolumes/particles in the datasets for the individual FSC curves are for panel **(a)** 40,962 (C2-hexamer from tubes); 16,910 (C5 pentamer from polyhedrons); 3,348 (C2 hexamer from polyhedrons); 1,877 (C3 hexamer from polyhedrons).
For panel (**b**) 69,700 (pentamer-hexamer 1”); 3,945 (pentamer-hexamer 1”2”); 4,099 (pentamer-hexamer 1”6”); 4,867 (pentamer-pentamer 1’1”); 52,548 (hexamer-hexamer 6’); 15,152 (hexamer-hexamer 2’6’); 4,128 (hexamer-hexamer 3’3”); 3,086 (hexamer-hexamer 3’4”); 3,546 (hexamer-hexamer 3’5”); 2,408 (hexamer-hexamer 4’4”); 3,755 (hexamer-hexamer 4’5”); 3,972 (hexamer-hexamer 5’5”); 7,071 (hexamer-hexamer 3'α); 12,640 (hexamer-hexamer 3'β); 2,202 (hexamer-hexamer 3'γ); 3,234 (hexamer-hexamer 4'α); 10,709 (hexamer-hexamer 4'β); 8,702 (hexamer-hexamer 4'γ); 3,195 (hexamer-hexamer 5'α); 3,618 (hexamer-hexamer 5'β); 15,133 (hexamer-hexamer 5'γ). For panel (**c**) 21,498 (T=1 CA); 406 (T=3 CA).

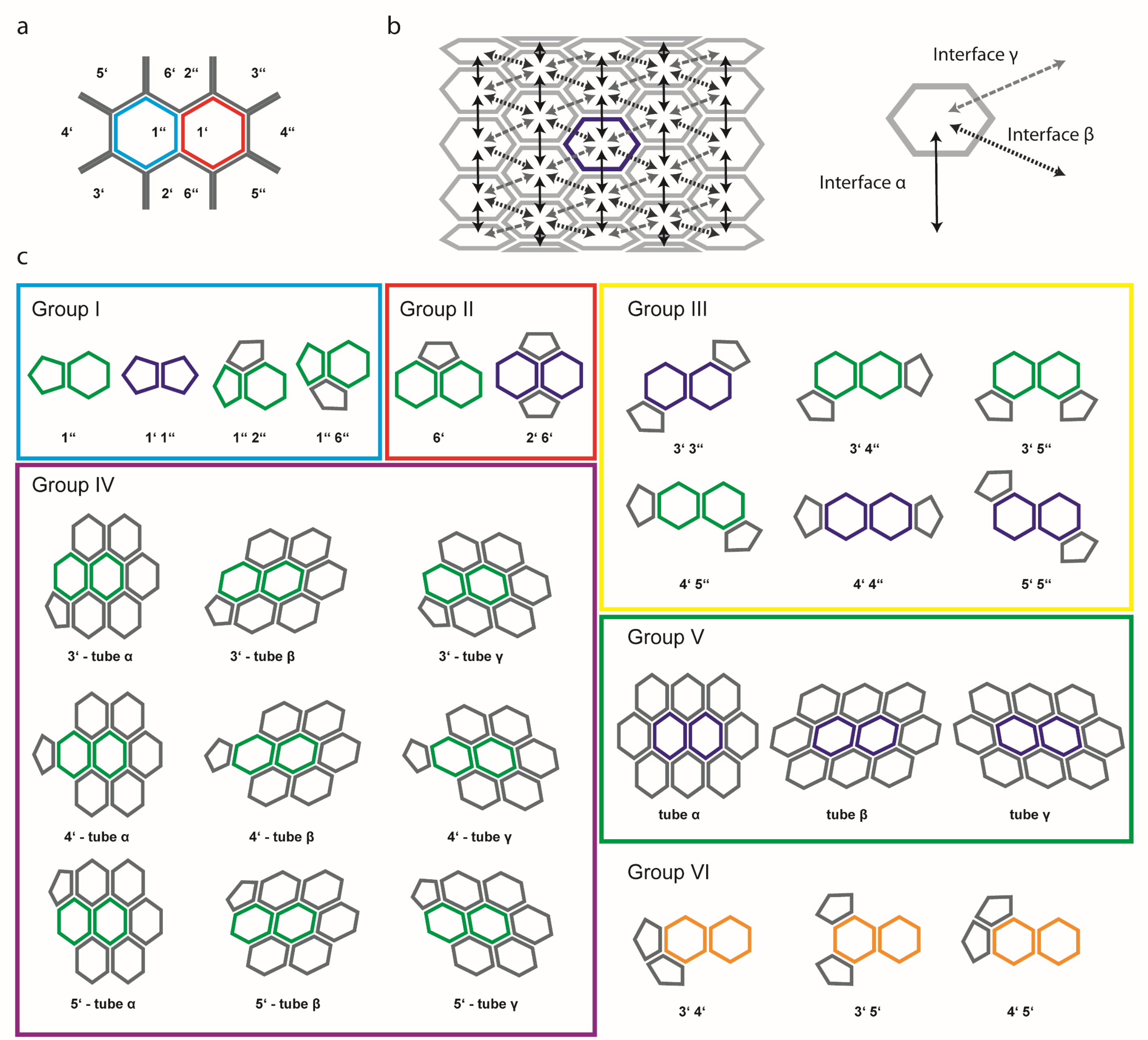

Supplementary Figure 4: Context-based classification. Graphical summary of the basic principles underlying the context-based classification. **a)** The numbering scheme for classification. Numbering of a unit pair is derived from the position of pentamer with respect to the two units of a unit pair; the central unit is colored cyan and any pentamers neighboring with this unit are marked with a single prime; the adjacent unit of the pair is colored red and any neighboring pentamers are marked with a double prime. **b)** The three different hexamer-hexamer connections in a regular CASPNC tube are shown. The tube axis in the scheme is horizontal. Connections α, β, and γ are shown in solid black, short-dashed dark grey, and long-dashed light grey arrows, respectively. These hexamer-hexamer connections are used to subclassify Groups 4 and 5. **c)** Schematic depiction of different classes distinguished during context-based classification. Groups of classes with related context are highlighted by a colored frame, as in Fig. 3: cyan: group I; red: group II; yellow: group III; purple: group IV; green: group IV. Hexagons and pentagons represent CA hexamers and pentamers, respectively. Two hexamers and pentamers form a unit pair, a basic unit, which is a subject to classification. The unit pairs colored blue consist of two units with identical geometric context based on the classification criteria. The unit pairs colored green consist of two units with different geometric context based on the classification criteria, resulting in two classes that are related by a 180 degree rotation during consensus alignment. In case of the orange unit pairs from Group VI the context of one unit is neglected, as it would lead to too sparsely populated classes. Therefore, group VI was used only during consensus alignment, but not for generating the final bin1 maps.

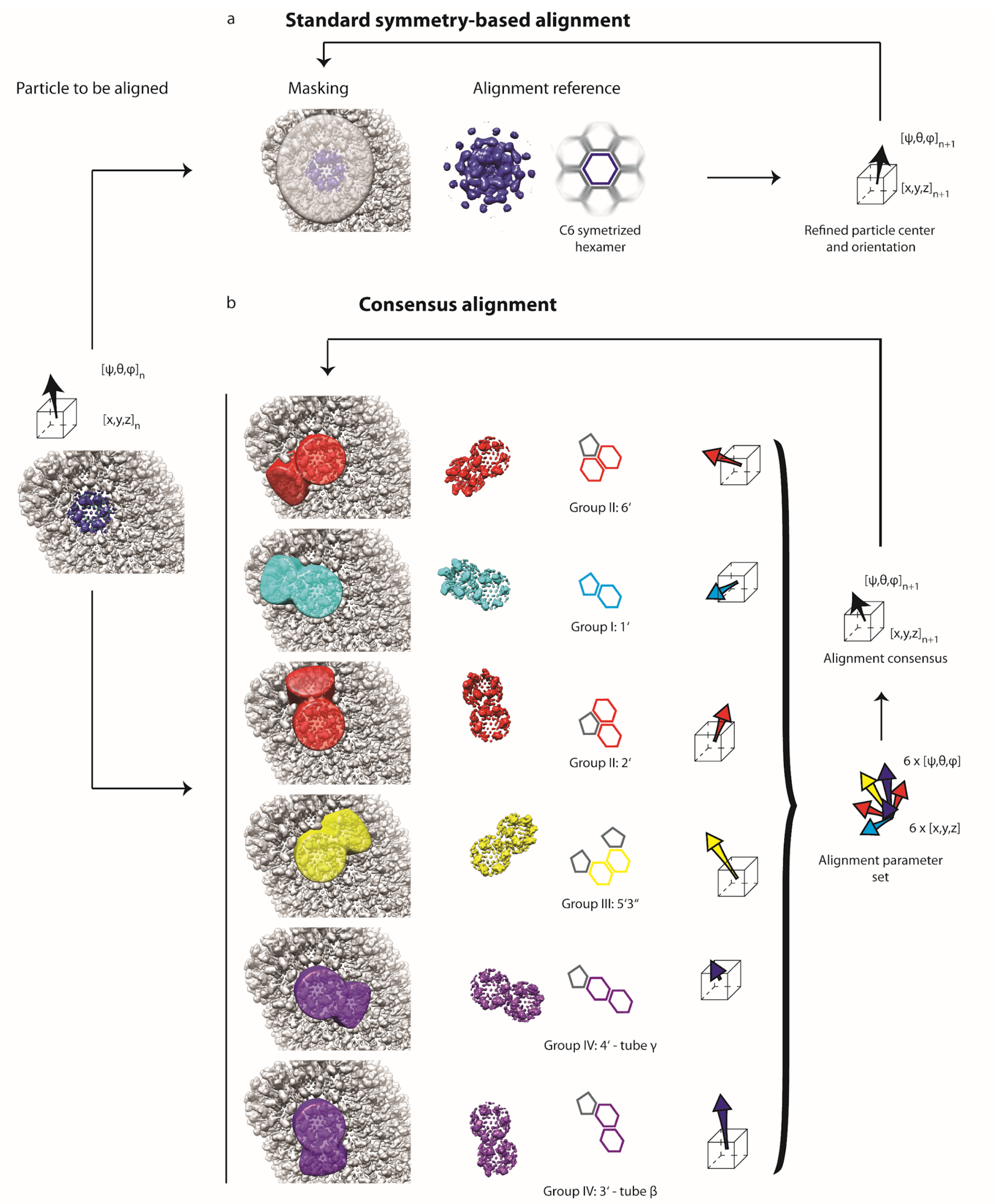

Supplementary Figure 5: Graphical summary of the consensus alignment. a) Alignment of CA hexamers using the "standard" approach: from left to right – a single hexamer in the context of a grey VLP is highlighted in

blue; the subvolume containing this hexamer is masked and iteratively aligned against a symmetrized reference, refining translation and orientation parameters of the subvolume. **b)** The consensus alignment builds on the same principles as the “standard” alignment routine. However, the alignment is performed for each unit multiple times equal to the number of its adjacent units. Each pair of the central and adjacent unit are classified according to their context (see groups in Supplementary Figure 4), and a reference representing the respective class is used for alignment in each case, while applying a tight mask around the central and the respective adjacent unit. Therefore, in each round multiple alignment parameters per unit are obtained. The consensus of the individual alignments per unit yields the alignment parameters for the next iteration.

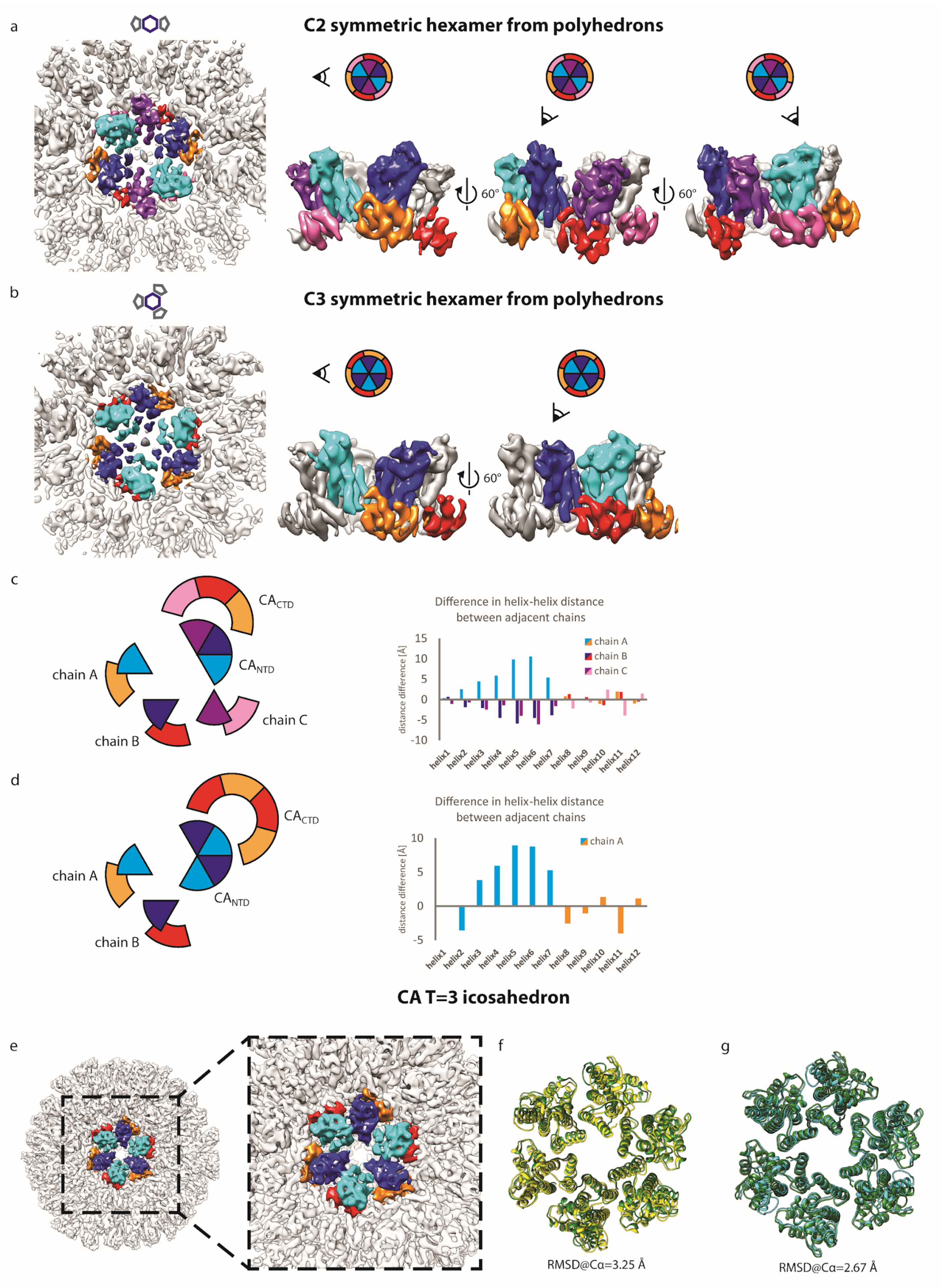

a
C2 symmetric hexamer from polyhedrons
60°
60°
b
C3 symmetric hexamer from polyhedrons
60°
c
CA$_{CTD}$
CA$_{NTD}$
chain A
chain B
chain C
Difference in helix-helix distance between adjacent chains
distance difference [Å]
chain A
chain B
chain C
15
10
5
0
-5
-10
helix1 helix2 helix3 helix4 helix5 helix6 helix7 helix8 helix9 helix10 helix11 helix12
d
CA$_{CTD}$
CA$_{NTD}$
chain A
chain B
Difference in helix-helix distance between adjacent chains
distance difference [Å]
chain A
10
5
0
-5
helix1 helix2 helix3 helix4 helix5 helix6 helix7 helix8 helix9 helix10 helix11 helix12
CA T=3 icosahedron
e
f
RMSD@Cα=3.25 Å
g
RMSD@Cα=2.67 Å

Supplementary Figure 6: The RSV CA pentamer structurally distorts adjacent hexamers. a) Distortion of a CA hexamer flanked by two pentamers in polyhedral VLPs. Left: EM-density of the C2-symmetric hexamer surrounded by two pentamers, shown in top view; middle and right: side views showing neighboring symmetry-independent CA monomers. Symmetry-independent CA monomers are distinguished by colors: cyan/orange, blue/red, and purple/pink, respectively. The viewing angle is indicated by an eye symbol. The two hexamer CA_{NTD}'s facing the pentamers show a larger opening. **b)** Distortion of a CA hexamer flanked by three pentamers in polyhedral VLPs. Views are identical to A). Side views are showing neighboring symmetry-independent CA monomers in cyan/orange, and blue/red, respectively. Again, the separation of the hexamer CA_{NTD}'s facing the pentamers is clearly visible. **c-d)** Distortion analysis of C2-, and C3-symmetric CA hexamers, respectively. Left: schematic view of CA hexamer showing symmetry-independent CA copies. The color code is as in (a and b). Right: difference in distance between identical helices of two adjacent CA copies measured per helix and symmetry-independent CA copy. **e-g)** T=3 CA icosahedron. **e)** Isosurface representation of a T=3 icosahedron solved by single particle analysis cryo-EM; the dashed line highlights one hexamer colored identically as in panel B, with two symmetry independent CA copies colored differently. **f)** The T=3 icosahedron hexamer CA model obtained via rigid body fitting of the individual CA domains separately (green) into the EM-density. The identical model is rotated by 60° (yellow) and shown in superimposition. The RMSD of 3.25 Å shows that the T=3 icosahedron hexamer is not 6-fold symmetric. **g)** Comparison of the CA hexamer model from T=3 icosahedrons (green) and of a C3-symmetric CA hexamer from polyhedrons made by rigid body fitting of the individual CA domains separately (cyan). The RMSD of 2.67 Å indicates that both models are distorted by the presence of adjacent pentamers, but the distortion is less apparent in the T=3 icosahedra.

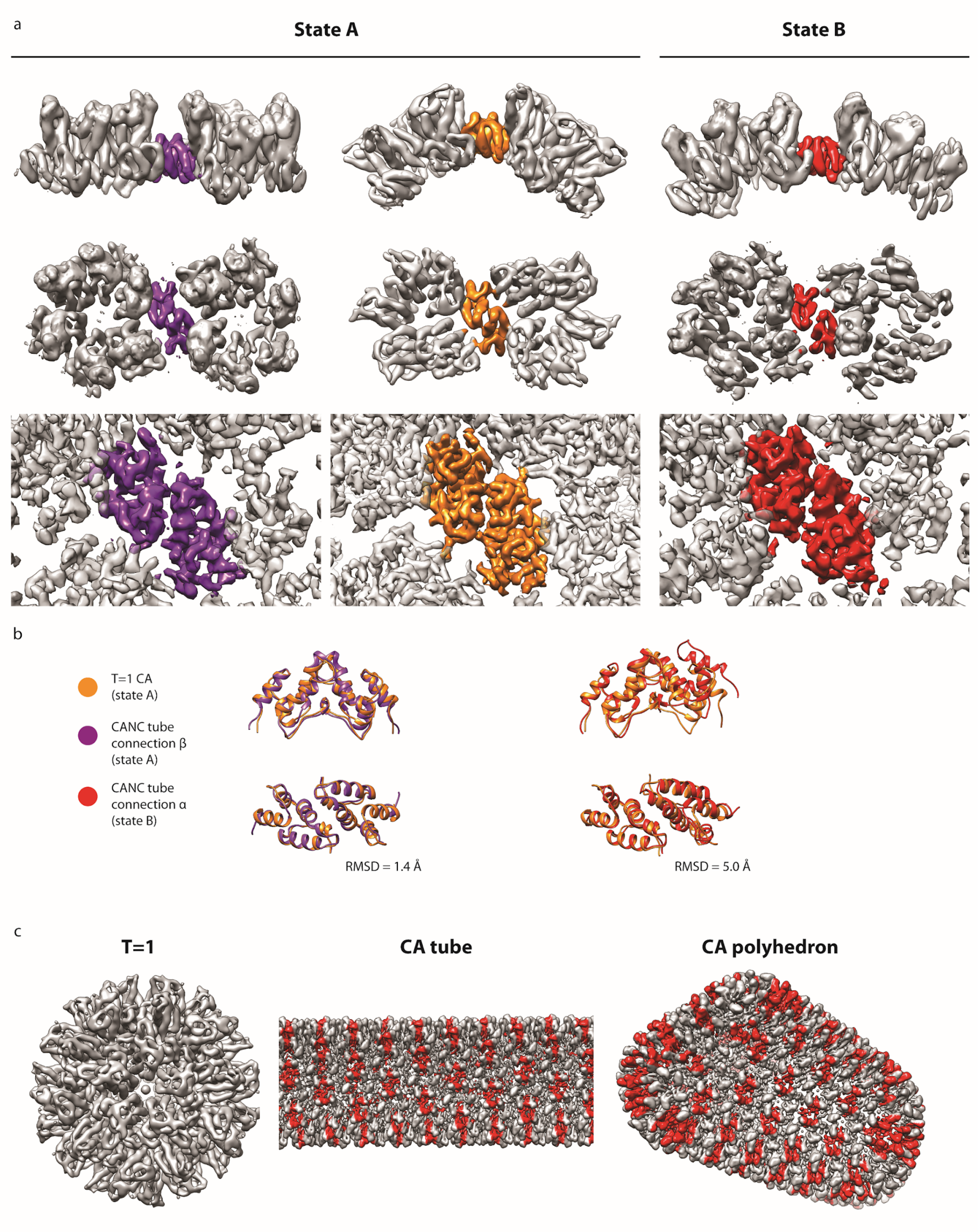

Supplementary Figure 7: Two states of RSV CA_{CTD} dimer interface. a) Two distinct forms of CA_{CTD} dimer (as determined by clustering of pairwise RMSD measurements) were observed in RSV VLPs. State B is found only

in high curvature hexamer-hexamer interfaces, whereas state A is found in pentamer-pentamer, pentamer-hexamer, and low-curvature hexamer-hexamer interfaces. Top and middle rows: cryoEM maps showing two adjacent hexa/pentamers filtered to 8 Å from side and top view, respectively; bottom row: cryoEM maps filtered to the resolution determined at 0.143 criterion. Two adjacent CA_{CTD}s are colored according to the following color code: purple – tube connection β (hexamer-hexamer); orange – T=1 CA (pentamer-pentamer); red – tube connection α (hexamer-hexamer). **b)** Real-space refined models are overlaid in order to illustrate the difference between state A and state B dimer, and similarity of state A dimers found in different VLPs. Left: comparison of two state A CTD dimers from CASPNC tube connection β (purple), and T=1 CA (orange); right: comparison of state A CTD dimer from T=1 CA (orange) and CASPNC tube – connection α (red). **c.** The two states are highlighted on different shapes of RSV VLPs: grey – state A; red – state B.

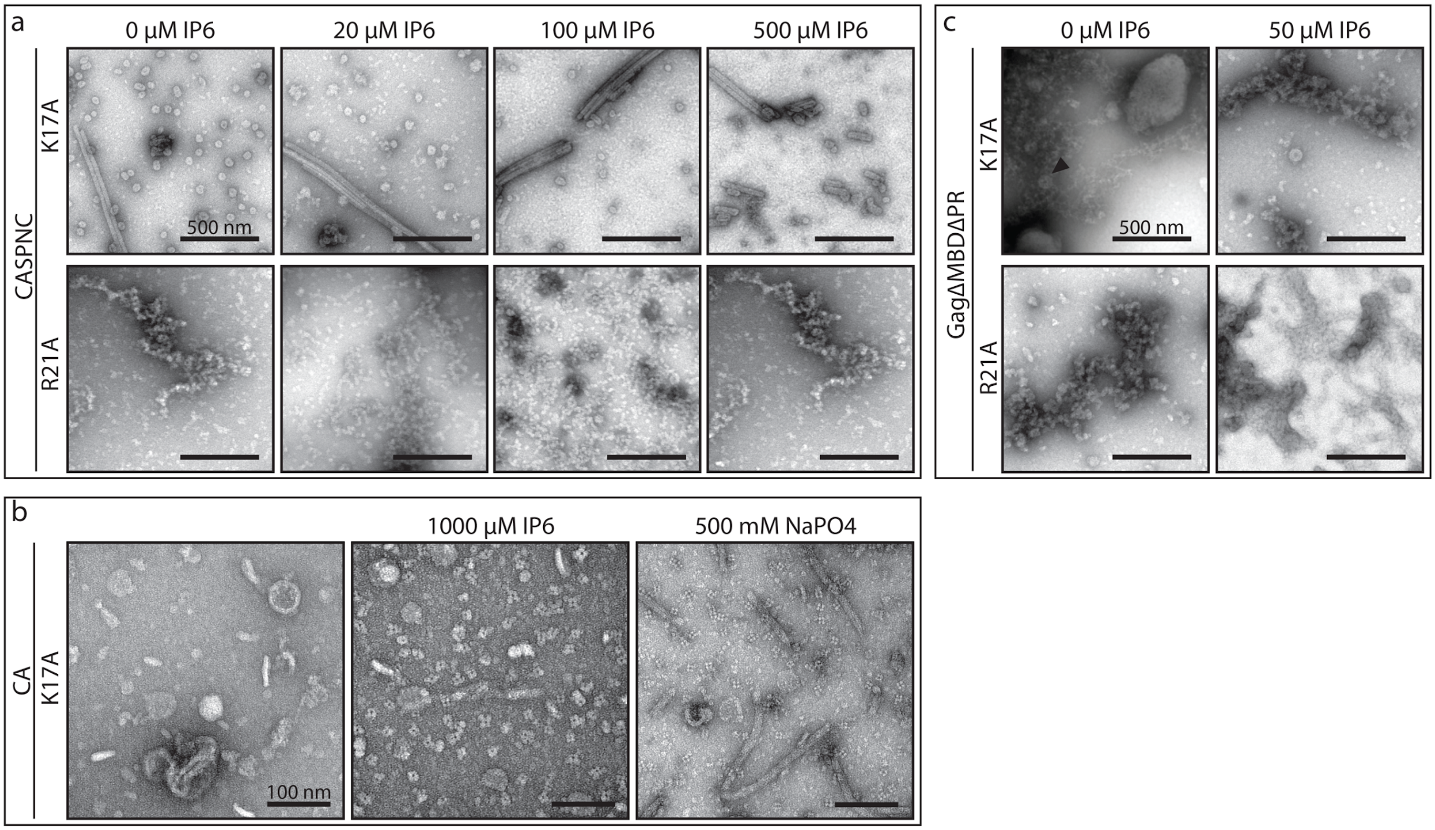

Supplementary Figure 8: K17A and R21A mutant assemblies. a) CASPNC K17A or R21A mutants assembled with increasing concentrations of IP6. **b)** CA K17A protein without and with IP6 or $NaPO_4$. **c)** Assembly of GagΔMBDΔPR K17A or R21A mutants without or with IP6

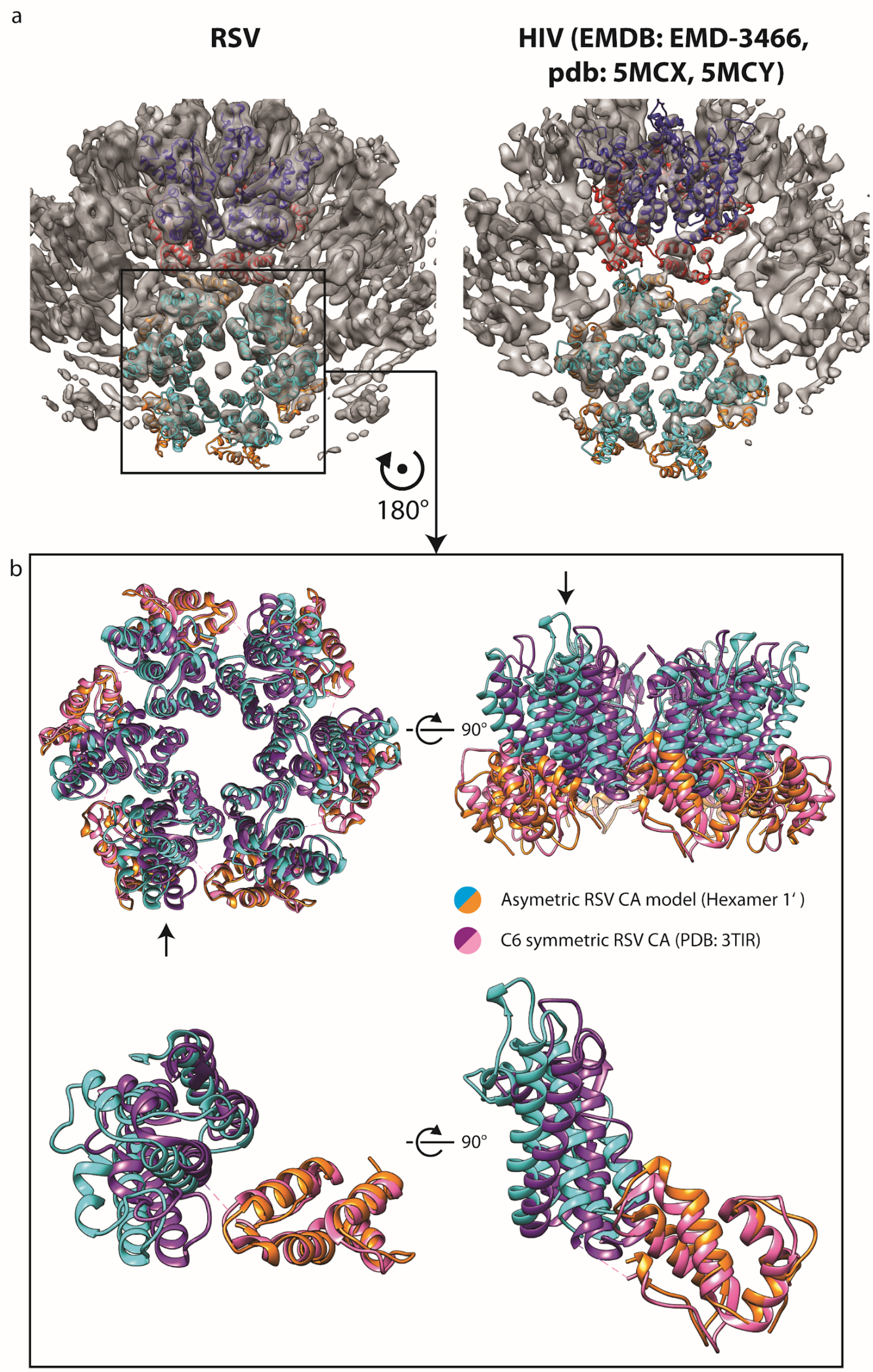

Supplementary Figure 9: CA hexamer flexibility in RSV and HIV-1. a) Isosurface representation of the RSV (left) and HIV-1 (right) CA pentamer and its surroundings with models of the CA pentamer and CA hexamer fitted into the density (EMDB and PDB codes for the HIV-1 structure [9] and model are annotated). Note that the RSV CA pentamer induces an opening in the adjacent CA hexamer. **b)** Comparison of the models of RSV CA hexamer derived by subtomogram averaging and X-ray crystallography [22]. Cyan/orange – asymmetric model obtained by rigid body fitting of individual CA domains into the cryo-electron microscopy density of the hexamer adjacent to a pentamer; purple/pink – C6 symmetric X-ray crystallographic model (pdb 3TIR). The hexamer and one isolated monomer (annotated with an arrow) are shown in top and side views.

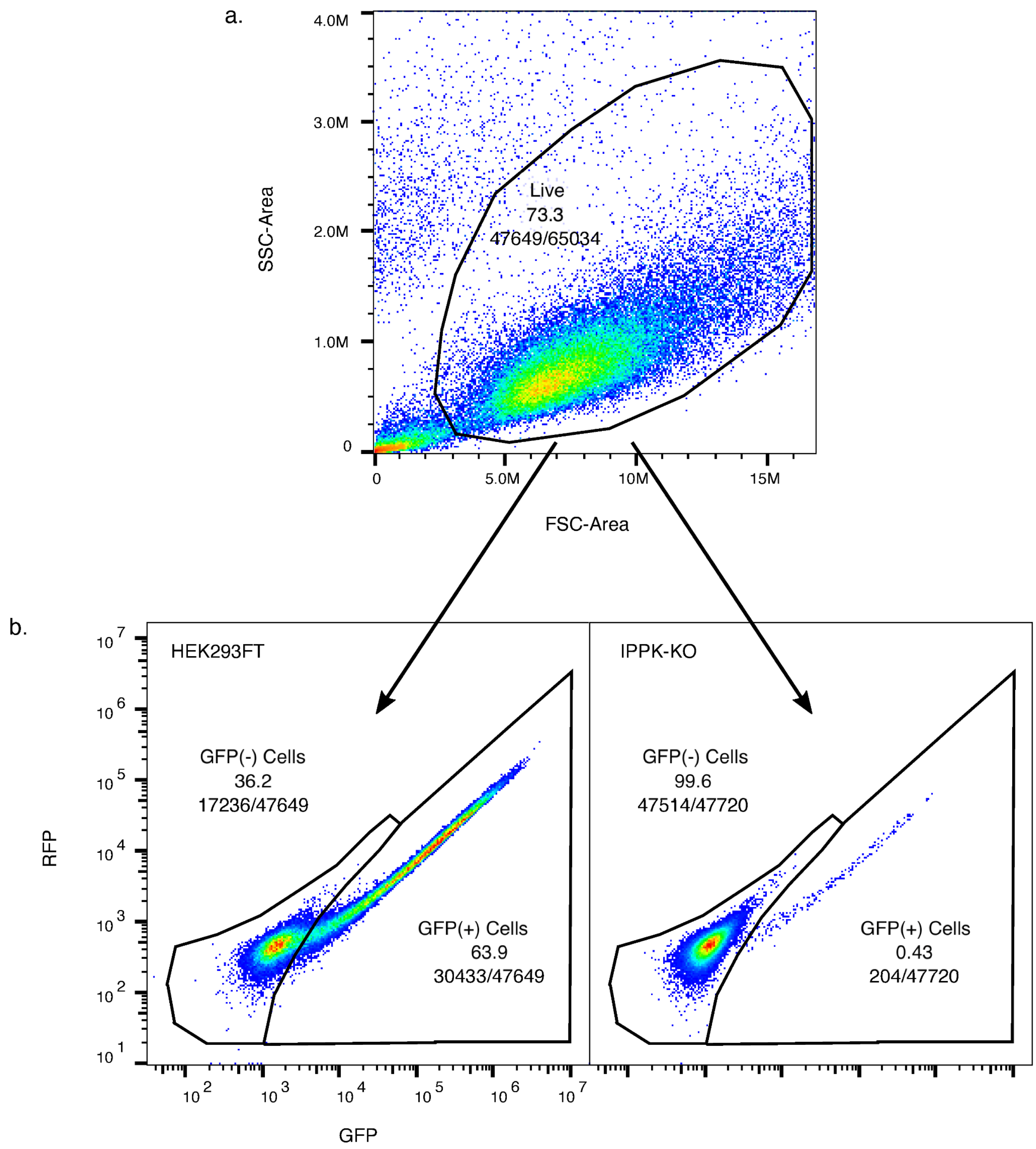

Supplementary Figure 10: Flow cytometry gating strategy. a) Events were plotted along the forward (FSC) and the side scatter (SSC) x-axis and y-axis respectively. Live cells were gated for the correct size and morphology for further sorting. **b)** Live cells were plotted along GFP and RFP on the x-axis and y-axis respectively. Cells not expressing GFP, GFP(-), and cells expressing GFP, GFP(+), were gated accordingly. Left, a representative plot of virus titer from an RSV-GFP provirus produced in WT HEK293FTs. Right, a representative plot of virus titer from an RSV-GFP provirus produced in IPPK-KO HEK293FTs.

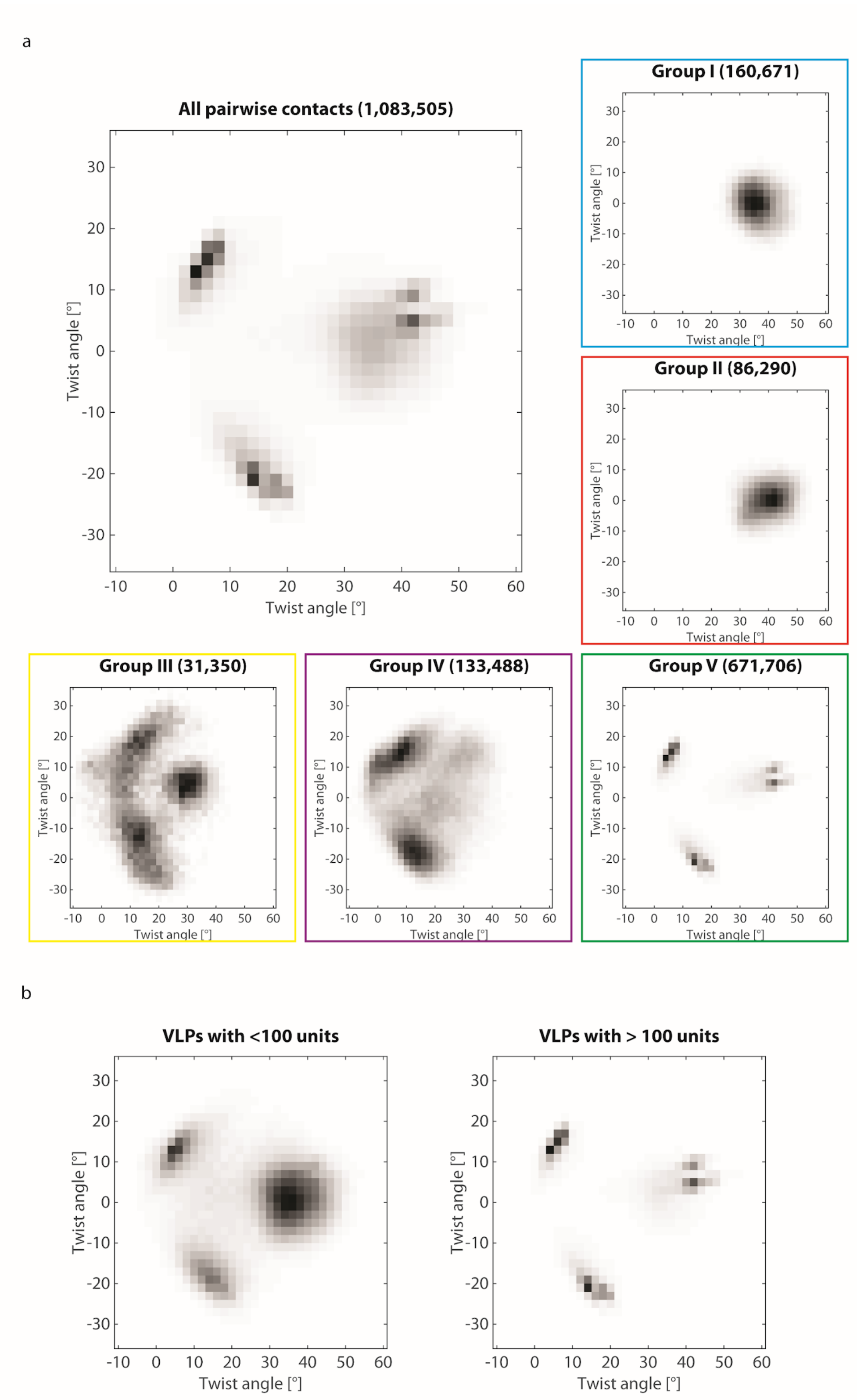

Supplementary Figure 11: Flexibility of RSV CASPNC VLPs. Tilt-twist plots were used to examine flexibility of unit pairs. Since neither tilt nor twist angles were used as criteria during classification, they could be utilized to assess the quality of classification and progress of subtomogram alignment. **a)** Large panel: all pairwise contacts between adjacent units in the CASPNC dataset; small panels: contacts between unit pairs belonging to the different groups. Coloring is as in Figure 3 and Supplementary Figure 4. Numbers in brackets for the individual plots describe their total unit pair numbers. **b)** Tilt-twist analysis plots for VLPs with less or more than 100 units are shown on the left and right side, respectively. Note that large VLPs consist mostly of group V – tubular unit connections.

Dataset		RSV CASPNC STA	RSV CA T=1 and T=3 SPA
**Acquisition settings**	**Microscope**	FEI Titan Krios	FEI Talos Arctica
	**Voltage (keV)**	300	200
	**Detector**	Gatan Quantum K2	Gatan Quantum K3
	**Energy-filter**	Yes	Yes
	**Slit width (eV)**	20	20
	**Super-resolution Mode**	Yes	Yes
	**Å/pixel**	1.328	1.24
	**Defocus range (μm)**	-1.5 to 4	-0.8-1.5
	**Defocus step (μm)**	0.25	0.1
	**Acquisition scheme**	-66/63°, 3°, SerialEM	Not applicable
	**Total dose (electrons/ Å^2)**	~156	~50
	**Dose rate (electrons/ Å^2/sec)**	~2.5	~32
	**Frame number**	10	50
	**Tomogram/micrograph number**	49	2394

Supplementary Table 1: Data acquisition statistics

Data	VLPs	Asymmetric units	Resolution (Å)
**Full RSV CASPNC dataset**	**2,595**		
**C2 hexamer from tubes**	**236**	81,924	4.3
**C5 pentamer from polyhedrons**		84,550	5.8
**C2 hexamer from polyhedrons**		6,696	7.4
**C3 hexamer from polyhedrons**		5,631	7.5
**Asymmetric dimers - 21 classes**		237,710 (2,202 – 69,700 per class)	6.0 – 9.1

Supplementary Table 2: Subtomogram averaging data set statistics
