# Supplementary material for: Structure of the mature Rous sarcoma virus lattice reveals a role for IP6 in the formation of the capsid hexamer
Source: Nat Commun. 2021 May 28;12:3226. doi: 10.1038/s41467-021-23506-0 (PMC8163826; doi:10.1038/s41467-021-23506-0)
Supplement: Supplementary file 7 — Description of Additional Supplementary Files [file 41467_2021_23506_MOESM7_ESM.pdf]

Movie S1

Mature RSV CASPNC tubular and polyhedral VLPs. Animated Z-slices through an example tomogram. CA hexamers and pentamers in the tomogram are visualized by cyan hexagons and red pentagons, respectively.

Movie S2

3D visualization of the structure of RSV CA hexamer derived from tubular VLPs. Video of the low-pass filtered composite map of RSV CASPNC tube, the 4.3 Å structure of RSV CA hexamer and the refined model (CA_{NTD} – cyan, CA_{CTD} – orange).

Movie S3

Morph between hexamer – pentamer, and two hexamers sharing a pentamer neighbor. Two adjacent CA_{NTD} domains of one hexamer are colored in blue and cyan. Note the amount of movement between the highlighted domains induced by accommodating the pentamer.

Movie S4

3D visualization of a composite map of the polyhedral VLP shown in Fig. 3. The map was built by placing the averages for the respective classes into the positions determined by subtomogram alignment. The color code corresponds to Fig. 3. The structure of pentamer-hexamer interface solved at 6.0 Å resolution is shown together with the rigid-body fitted model (CA_{NTD} – cyan, CA_{CTD} – orange).

Movie S5

Morph between pentamer-pentamer interface from CA T=1 icosahedron and low-tilt hexamer-hexamer interface from CASPNC tubes. The color-highlighted interface is stationary compared to the rest of the structure as morphing between the two cryo-EM maps.
