# Supplementary material for: Structure of the mature Rous sarcoma virus lattice reveals a role for IP6 in the formation of the capsid hexamer
Source: Nat Commun. 2021 May 28;12:3226. doi: 10.1038/s41467-021-23506-0 (PMC8163826; doi:10.1038/s41467-021-23506-0)
Supplement: Supplementary file 8 — Reporting Summary [file 41467_2021_23506_MOESM8_ESM.pdf]

nature research

Corresponding author(s): Robert A. Dick

Last updated by author(s): Apr 17, 2021

Reporting Summary

Nature Research wishes to improve the reproducibility of the work that we publish. This form provides structure for consistency and transparency in reporting. For further information on Nature Research policies, see our Editorial Policies and the Editorial Policy Checklist.

Statistics

For all statistical analyses, confirm that the following items are present in the figure legend, table legend, main text, or Methods section.

n/a	Confirmed	
☐	☒	The exact sample size (*n*) for each experimental group/condition, given as a discrete number and unit of measurement
☐	☒	A statement on whether measurements were taken from distinct samples or whether the same sample was measured repeatedly
☒	☐	The statistical test(s) used AND whether they are one- or two-sided<br>*Only common tests should be described solely by name; describe more complex techniques in the Methods section.*
☒	☐	A description of all covariates tested
☒	☐	A description of any assumptions or corrections, such as tests of normality and adjustment for multiple comparisons
☐	☒	A full description of the statistical parameters including central tendency (e.g. means) or other basic estimates (e.g. regression coefficient) AND variation (e.g. standard deviation) or associated estimates of uncertainty (e.g. confidence intervals)
☒	☐	For null hypothesis testing, the test statistic (e.g. *F*, *t*, *r*) with confidence intervals, effect sizes, degrees of freedom and *P* value noted<br>*Give P values as exact values whenever suitable.*
☒	☐	For Bayesian analysis, information on the choice of priors and Markov chain Monte Carlo settings
☒	☐	For hierarchical and complex designs, identification of the appropriate level for tests and full reporting of outcomes
☒	☐	Estimates of effect sizes (e.g. Cohen's *d*, Pearson's *r*), indicating how they were calculated

Our web collection on statistics for biologists contains articles on many of the points above.

Software and code

Policy information about availability of computer code

Data collection	Data collection: Digital Micrograph (as included in the Gatan Microscopy suite version 3.22.1435.1) and SerialEM (3.7.0 beta), FEI AutoCTF (0.6.10)
Data analysis	Tilt series and tomogram visualisation: IMOD (4.9) and 3dmod (part of the IMOD software package)<br>Subtomogram averaging: Dynamo (1.1.133), TOM toolbox (version numbers do not exist for this software), AV3 (version numbers do not exist for this software)<br>In-house developed analysis scripts: MATLAB scripts for context-based classification and consensus alignments, as well as documentation on their usage are available for download at https://schurlab.ist.ac.at/downloads/<br>Accuri - Csampler Software Version 1.0.264.21

For manuscripts utilizing custom algorithms or software that are central to the research but not yet described in published literature, software must be made available to editors and reviewers. We strongly encourage code deposition in a community repository (e.g. GitHub). See the Nature Research guidelines for submitting code & software for further information.

Data

Policy information about availability of data

All manuscripts must include a data availability statement. This statement should provide the following information, where applicable:

- Accession codes, unique identifiers, or web links for publicly available datasets
- A list of figures that have associated raw data
- A description of any restrictions on data availability

The electron microscopy density maps and the corresponding models and a representative tomogram have been deposited in the Electron Microscopy Data Bank (accession codes: EMD-12485 [https://www.ebi.ac.uk/pdbe/entry/emdb/EMD-12485], EMD-12486 [https://www.ebi.ac.uk/pdbe/entry/emdb/EMD-12486], EMD-12487 [https://www.ebi.ac.uk/pdbe/entry/emdb/EMD-12487], EMD-12488 [https://www.ebi.ac.uk/pdbe/entry/emdb/EMD-12488], EMD-12489 [https://www.ebi.ac.uk/pdbe/entry/emdb/EMD-12489], EMD-12490 [https://www.ebi.ac.uk/pdbe/entry/emdb/EMD-12490], EMD-12491 [https://www.ebi.ac.uk/pdbe/entry/emdb/EMD-12491], EMD-12492 [https://www.ebi.ac.uk/pdbe/entry/emdb/EMD-12492], EMD-12493 [https://www.ebi.ac.uk/pdbe/entry/emdb/EMD-12493], EMD-12494 [https://www.ebi.ac.uk/pdbe/entry/emdb/EMD-12494], EMD-12495 [https://www.ebi.ac.uk/pdbe/entry/emdb/EMD-12495], EMD-12496 [https://www.ebi.ac.uk/pdbe/entry/emdb/EMD-12496], EMD-12497 [https://www.ebi.ac.uk/pdbe/entry/emdb/EMD-12497], EMD-12498 [https://www.ebi.ac.uk/pdbe/entry/emdb/EMD-12498], EMD-12499 [https://www.ebi.ac.uk/pdbe/entry/emdb/EMD-12499], EMD-12500 [https://www.ebi.ac.uk/pdbe/entry/emdb/EMD-12500], EMD-12501 [https://www.ebi.ac.uk/pdbe/entry/emdb/EMD-12501], EMD-12502 [https://www.ebi.ac.uk/pdbe/entry/emdb/EMD-12502], EMD-12503 [https://www.ebi.ac.uk/pdbe/entry/emdb/EMD-12503], EMD-12504 [https://www.ebi.ac.uk/pdbe/entry/emdb/EMD-12504], EMD-12505 [https://www.ebi.ac.uk/pdbe/entry/emdb/EMD-12505], EMD-12506 [https://www.ebi.ac.uk/pdbe/entry/emdb/EMD-12506], EMD-12507 [https://www.ebi.ac.uk/pdbe/entry/emdb/EMD-12507], EMD-12508 [https://www.ebi.ac.uk/pdbe/entry/emdb/EMD-12508], EMD-12509 [https://www.ebi.ac.uk/pdbe/entry/emdb/EMD-12509], EMD-12510 [https://www.ebi.ac.uk/pdbe/entry/emdb/EMD-12510], EMD-12511 [https://www.ebi.ac.uk/pdbe/entry/emdb/EMD-12511], EMD-12774 [https://www.ebi.ac.uk/pdbe/entry/emdb/EMD-12774]) and Protein Data Bank (accession codes: 7NO0 [https://doi.org/10.2210/pdb7NO0/pdb], 7NO1 [https://doi.org/10.2210/pdb7NO1/pdb], 7NO2 [https://doi.org/10.2210/pdb7NO2/pdb], 7NO3 [https://doi.org/10.2210/pdb7NO3/pdb], 7NO4 [https://doi.org/10.2210/pdb7NO4/pdb], 7NO5 [https://doi.org/10.2210/pdb7NO5/pdb], 7NO6 [https://doi.org/10.2210/pdb7NO6/pdb], 7NO7 [https://doi.org/10.2210/pdb7NO7/pdb], 7NO8 [https://doi.org/10.2210/pdb7NO8/pdb], 7NO9 [https://doi.org/10.2210/pdb7NO9/pdb], 7NOA [https://doi.org/10.2210/pdb7NOA/pdb], 7NOB [https://doi.org/10.2210/pdb7NOB/pdb], 7NOC [https://doi.org/10.2210/pdb7NOC/pdb], 7NOD [https://doi.org/10.2210/pdb7NOD/pdb], 7NOE [https://doi.org/10.2210/pdb7NOE/pdb], 7NOF [https://doi.org/10.2210/pdb7NOFpdb], 7NOG [https://doi.org/10.2210/pdb7NOG/pdb], 7NOH [https://doi.org/10.2210/pdb7NOH/pdb], 7NOI [https://doi.org/10.2210/pdb7NOI/pdb], 7NOJ [https://doi.org/10.2210/pdb7NOJ/pdb], 7NOK [https://doi.org/10.2210/pdb7NOK/pdb], 7NOL [https://doi.org/10.2210/pdb7NOL/pdb], 7NOM [https://doi.org/10.2210/pdb7NOM/pdb], 7NON [https://doi.org/10.2210/pdb7NON/pdb], 7NOO [https://doi.org/10.2210/pdb7NOO/pdb], 7NOP [https://doi.org/10.2210/pdb7NOP/pdb], 7NOQ [https://doi.org/10.2210/pdb7NOQ/pdb]), respectively. The pdb we used as a starting model to refine RSV CA into our structure was PDB 3TIR [https://doi.org/10.2210/pdb3TIR/pdb].

Field-specific reporting

Please select the one below that is the best fit for your research. If you are not sure, read the appropriate sections before making your selection.

☒ Life sciences ☐ Behavioural & social sciences ☐ Ecological, evolutionary & environmental sciences

For a reference copy of the document with all sections, see nature.com/documents/nr-reporting-summary-flat.pdf

Life sciences study design

All studies must disclose on these points even when the disclosure is negative.

Sample size	Flow data: The standard practice of increasing sample until a maximum power level of 100% was achieved.<br>Sample-size calculation was not performed prior to cryo-ET acquisition. Sample-size was considered large enough, since processing yielded structures with sufficient resolution for unambiguous fitting of the protein backbones. A total of 49 tilt-series were processed for the study.
Data exclusions	Where applicable, data was not excluded.
Replication	Where applicable, replications were successful.
Randomization	Randomization was not applicable.
Blinding	Blinding was not applicable to cryo EM data.

Reporting for specific materials, systems and methods

We require information from authors about some types of materials, experimental systems and methods used in many studies. Here, indicate whether each material, system or method listed is relevant to your study. If you are not sure if a list item applies to your research, read the appropriate section before selecting a response.

Materials & experimental systems

n/a	Involved in the study
☐	☒ Antibodies
☐	☒ Eukaryotic cell lines
☒	☐ Palaeontology and archaeology
☒	☐ Animals and other organisms
☒	☐ Human research participants
☒	☐ Clinical data
☒	☐ Dual use research of concern

Methods

n/a	Involved in the study
☒	☐ ChIP-seq
☐	☒ Flow cytometry
☒	☐ MRI-based neuroimaging

Antibodies

Antibodies used	Rabbit anti-RSV-capsid; Goat anti-rabbit peroxidase-conjugated (Sigma, A0545); Mouse anti-GAPDH (Santa Cruz Biotechnology, SC-47724);Goat anti-mouse-HRP (Sigma, A5278).
Validation	Antibody validation is provided on the manufacturers websites, see below:<br>goat anti-rabbit peroxidase-conjugated antibody (Sigma, A0545) https://www.sigmaaldrich.com/catalog/product/sigma/a0545?lang=en&region=US<br>mouse anti-GAPDH antibody (Santa Cruz Biotechnology, SC-47724) https://www.scbt.com/p/gapdh-antibody-0411<br>goat anti-mouse-HRP (Sigma, A5278) https://www.sigmaaldrich.com/catalog/product/sigma/a5278?lang=en&region=US<br><br>Rabbit anti-RSV-capsid antibody was prepared in house and has been used in multiple publications verifying the specificity to the RSV CA domain. An example publication can be found at PMID: 11119594

Eukaryotic cell lines

Policy information about cell lines

Cell line source(s)	HEK 293T cells were purchased from Invitrogen (now ThermoFisher). A derivative of this cell line was generated by CRISPR-cas9 KO of the IPPK gene.
Authentication	Cell lines derived from the parent cell line were confirmed by amplifying and sequencing the target domain.
Mycoplasma contamination	Tested; no mycoplasma contamination
Commonly misidentified lines (See ICLAC register)	none: This cell line was used because it can be efficiently transfected and produces high titers of HIV-1 particles.

Flow Cytometry

Plots

Confirm that:

☒ The axis labels state the marker and fluorochrome used (e.g. CD4-FITC).

☒ The axis scales are clearly visible. Include numbers along axes only for bottom left plot of group (a 'group' is an analysis of identical markers).

☒ All plots are contour plots with outliers or pseudocolor plots.

☒ A numerical value for number of cells or percentage (with statistics) is provided.

Methodology

Sample preparation	HEK293T cells (Thermo, R70007) that were validated as contamination free were plated in 6 well format. After 6-16 hrs adherent cells were transfected using PEI and plasmid. Two-days post transfection, media containing virus was collected, frozen for a minimum of 1 hr, thawed, precleared (centrifugation at 3000xg for 5min), supernatant collected, and labeled as viral media. This viral media was added (transduced) in equal microliter amounts per sample to fresh HEK293T cells. Two-days post transduction, samples were lifted using Tryple-Express (Thermo, 12605028), fixed with 4-5% PFA, washed with PBS, and suspended in PBS. Cells were then collected on an Accuri-C6 Flow Cytometer, gating for green fluorescent cells.
Instrument	Accuri-C6
Software	Accuri - Csampler version 1.0.264.21
Cell population abundance	0.1%-70%

Gating strategy

Transduced cells turned green. We gated the green population. Gating the green cells is a score for infectivity.

☒ Tick this box to confirm that a figure exemplifying the gating strategy is provided in the Supplementary Information.
